# Open surgical treatment of subclavian artery pseudoaneurysm after endovascular repair: a case report

**DOI:** 10.1186/s13019-022-01775-0

**Published:** 2022-02-26

**Authors:** Kyo Seon Lee, Yochun Jung, In Seok Jeong, Sang Yun Song, Kook Joo Na, Sang Gi Oh

**Affiliations:** Department of Thoracic and Cardiovascular Surgery, Chonnam National University Hospital, Chonnam National University School of Medicine, 42, Jebong-ro, Dong-gu, Gwangju, 15772 South Korea

**Keywords:** Aneurysm, Endovascular treatment, Subclavian artery

## Abstract

**Background:**

Subclavian artery aneurysms are rare but may cause life-threatening complications. Surgical repair has been performed as a treatment of choice, but recently, with the development of endovascular treatment, many endovascular repairs have been performed to prevent surgical complications.

**Case presentation:**

A patient undergoing endovascular repair with a subclavian artery aneurysm was diagnosed with a type II endoleak with an enlarged aneurysmal sac. Surgical repair was performed to remove the aneurysmal sac compressing the adjacent organs.

**Conclusions:**

The highly mobile subclavian artery has abundant collaterals. Therefore, regular follow-up is essential for endovascular repair. Surgical repair is effective when adjacent organs are compressed by the aneurysm sac.

## Background

Subclavian artery aneurysm is an extremely rare disease that develops in < 1% of all aneurysms. Previously, true aneurysm caused by atherosclerosis or thoracic outlet syndrome was the main cause, but recently, traumatic pseudoaneurysm has been the most common cause [1, 2]. Surgical treatment has been established as the standard treatment for subclavian aneurysm, but currently, less invasive endovascular repair has replaced surgical repair [3]. However, caution should be taken as many collateral vessels and the highly mobile subclavian artery can cause endoleak. Herein, we present a case of surgical treatment for a type II endoleak that occurred after endovascular repair.

## Case presentation

A 56-year-old man presented with a palpable mass in the right neck. The patient did not know exactly when the mass was recognized but complained that it appeared to be larger than before. The boundaries of the mass were ambiguous, but pulsation was felt, and there was no tenderness. There were no specific findings except for a history of hepatic resection for trauma 25 years prior and cholecystectomy for gallstones 6 months prior. The patient had no history of trauma. The patient was hemodynamically stable, and there were no specific laboratory findings.

Neck computed tomography angiography (CTA) revealed proximal right subclavian artery aneurysm involving the vertebral artery (VA). The aneurysm was approximately 6 × 6 cm size with internal thrombus formation. Although the patient had no symptoms other than a palpable neck mass, surgical repair was recommended because the aneurysm was relatively large and had a thrombus inside. However, he did not feel any discomfort as the mass grew for a long time and there was no symptom of compression of other surrounding organs, so he preferred endovascular repair with less invasiveness than surgery. As the subclavian artery aneurysm started 2 cm after the bifurcation of the innominate artery, sufficient sealing with a stent graft was considered possible without the risk of a type 1 endoleak.

**Fig. 1:**
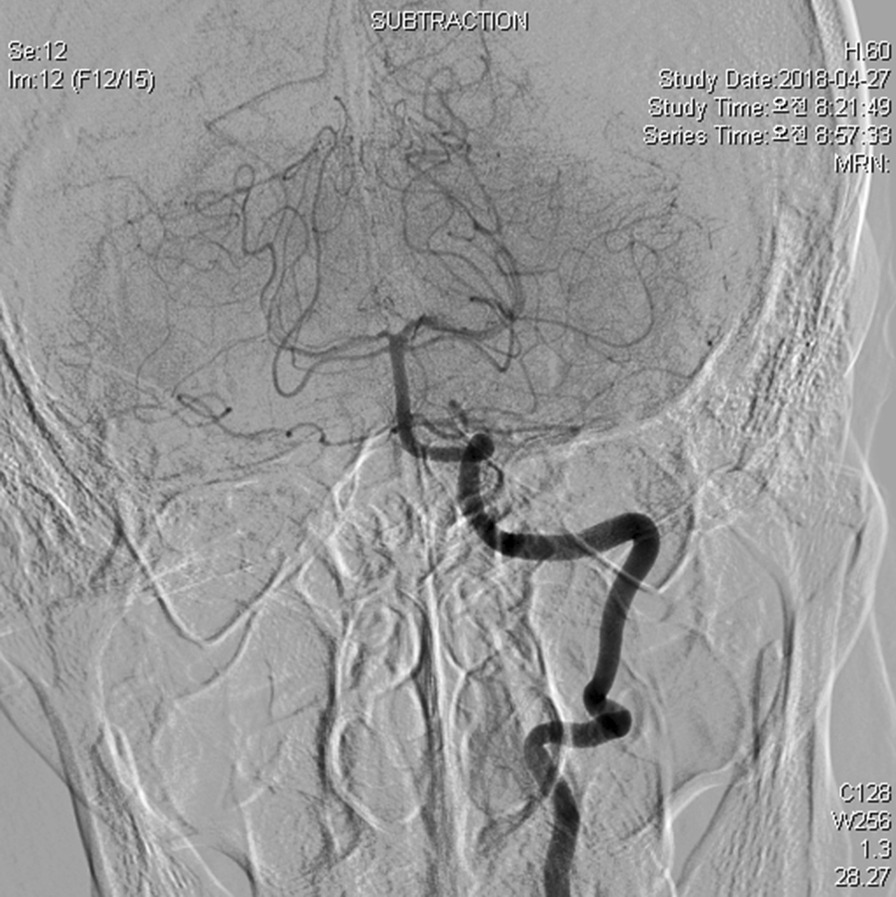
Cerebral angiography reveals dominant left vertebral artery

**Fig. 2:**
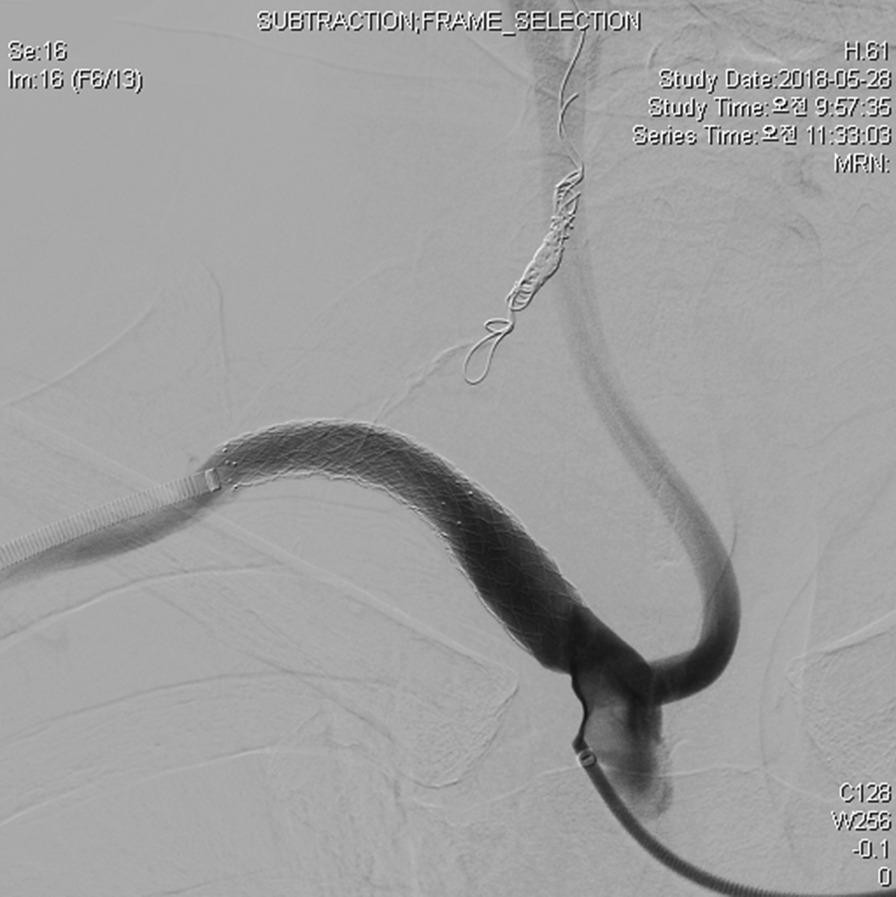
Stent-graft is inserted into the right subclavian artery. Moreover, the right vertebral artery is occluded by coil

First, it was confirmed that the collateral flow was well maintained even when the right VA was occluded on cerebral angiography through the left VA (Fig. 1). Subsequently, coil embolization was performed on the right VA, and two Lifestream 10 × 58 mm stents were inserted into the right subclavian artery, and a bare metal stent (Protege GPS 10 × 40 mm) was additionally inserted to prevent kinking (Fig. 2). The patient was discharged without any complications. Periodic follow-up was recommended to the patient, but he did not visit the outpatient clinic.

**Fig. 3:**
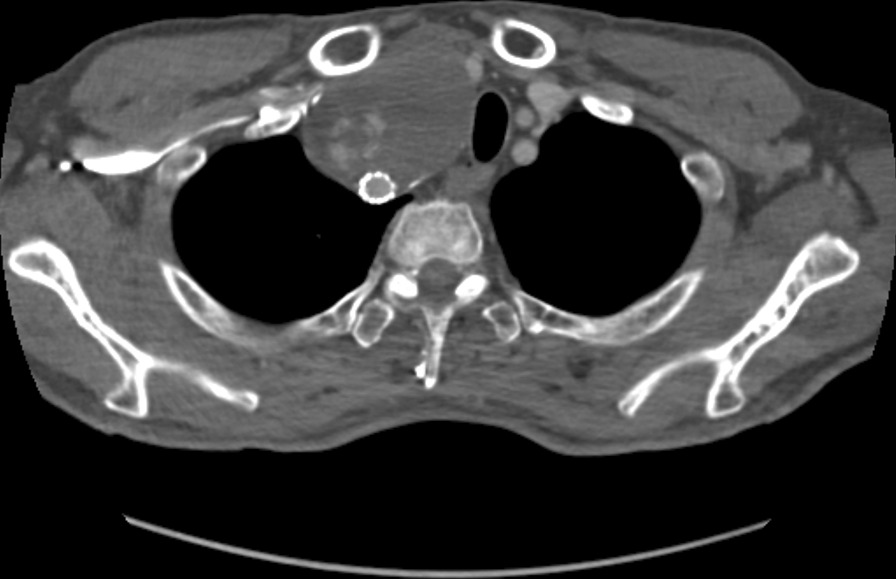
Type II endoleak was observed around the aneurysmal sac. The trachea is deviated to the left side due to aneurysm

After 2 years, the patient visited our hospital for hoarseness, which started 3 months prior. Neck CTA revealed that the aneurysmal sac with a maximum diameter of 7 cm, which was larger than it was 2 years ago due to type II endoleak (Fig. 3). The patient had dysphagia because the esophagus was partially compressed by the aneurysmal sac. Since the aneurysmal sac had to be removed, the patient was recommended to undergo surgical treatment, and he agreed.

**Fig. 4:**
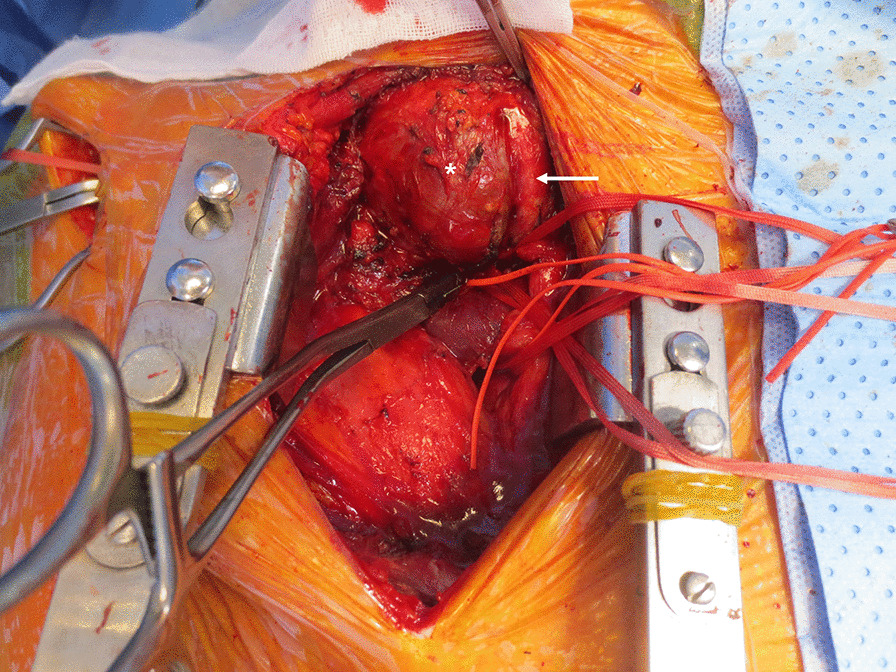
The aneurysm sac (*) is observed by partial sternotomy. The proximal right subclavian artery is clamped and the right common carotid artery (white arrow) is deviated. Additional incision is found in the right deltopectoral groove

After partial sternotomy through the right third intercostal space, the proximal innominate artery was exposed to isolate the right subclavian artery and right common carotid artery. The distal right subclavian artery was isolated using an additional incision of the right deltopectoral groove (Fig. 4).

After heparin administration, the proximal and distal subclavian arteries were clamped. After making an incision in the aneurysmal sac, the hematoma was removed. The main cause of the pressure effect was a hematoma within the sac; therefore, it was expected that the pressure effect would relieve after the hematoma was removed. Part of the stent-graft was exposed inside the aneurysmal sac, but the aneurysmal sac was obliterated without removing the stent-graft. Since the patency inside the stent-graft was well maintained and epithelialization was in progress, it was decided to keep the stent-graft rather than remove it and replace it with a new Dacron graft. The collateral vessels inside the sac that caused endoleak were sutured. The patient was discharged without any complications.

## Discussion

The subclavian artery is divided into proximal, middle, distal parts, according to its anatomical location. The proximal part is from its origin to the medial border of the anterior scalene muscle. At this site, the VA originates from the superior surface of the subclavian artery, the internal thoracic artery from its inferior surface, and the thyrocervical trunk from its anterior surface. The distal part is from the lateral border of the anterior scalene muscle to the lateral border of the first rib. The middle part is located between these two parts and at the dorsal part of the anterior scalene muscle [1].

There are different causes of aneurysm in the subclavian artery depending on the site. Atherosclerosis is the most common cause of aneurysm in the proximal part, collagen disorders in the middle part, and thoracic outlet syndrome in the distal part. However, trauma is the most common cause of all aneurysms regardless of the site due to the increasing number of percutaneous catheterizations recently [1, 2]. Even so, subclavian artery aneurysm is a rare disease with an extremely low incidence.

Symptoms of subclavian artery aneurysm vary depending on its location and size. In some cases, it is found by chance without symptoms or as a simple palpable mass on the neck. Local compression may cause dysphagia, hoarseness, or Horner’s syndrome. It can also cause ischemia of the arm or cerebral infarction due to embolism caused by internal thrombus. If proper treatment is not provided, rupture can occur. Although there are no clear guidelines for treatment, it is an indication for treatment if the patient has symptoms or a high risk for rupture or thromboembolism [1].

Subclavian artery aneurysm was traditionally treated surgically, but the part of the clavicle or sternum needs to be resected according to the location of the aneurysm. Moreover, in the process of manipulating the subclavian artery, injury to adjacent vessels or brachial plexus may occur. Recently, with the development of endovascular techniques, the use of less invasive stent-graft insertion has gradually increased. However, the subclavian artery has a risk of endoleak due to several branch vessels originating from each anatomical part. Furthermore, since the subclavian artery has several movements and passes through the first rib, stent kinking or fracture may occur [1, 4]. Particularly, it is important to determine the status of the internal thoracic artery and VA when treating aneurysms of the proximal part. The internal thoracic artery with a bypassed coronary artery must be revascularized. The VA converges into the basilar artery and supplies blood to the brainstem, cerebellum, and occipital lobes. However, in patients with thoracic aortic aneurysm, the patency cannot always be guaranteed because there are many variations [5]. Therefore, the patency of the VA must be checked through angiography preoperatively, and the dominancy VA must be revascularized [6]. In contrast, if the aneurysm contains a non-dominancy VA as in our case, the VA can be occluded by coil embolization to prevent endoleak [7]. Although cerebral angiography is invasive and has a risk of stroke, it is more useful than CTA in that it can directly check the blood flow and patency of the VA. However, the risk of stroke that can occur by performing cerebral angiography can sufficiently offset the risk of stroke that can occur by not checking the patency or distal cerebral blood flow.

Endoleak is an important complication of endovascular repair that requires additional intervention. Although intraoperative endoleak occurred in 6.1% of cases in a systematic review, the exact prevalence of endoleak for subclavian artery aneurysm is unknown [2]. Moreover, since type II endoleak is often detected during follow-up, the actual incidence of endoleak is estimated to be higher. The reason for the many endoleaks is thought to be that the subclavian artery has several movements and the upper limb arteries are rich in the collaterals. However, type II endoleak can also occur because of the surrounding small arteries, even if the branches of the subclavian artery are well managed. Therefore, periodic follow-up is essential because type II endoleaks may subsequently develop because of newly grown small vessels around the aneurysm [4]. In our case, the patient voluntarily avoided follow-up after endovascular repair, resulting in an enlarged aneurysmal sac by the surrounding small arteries. When the size of the aneurysmal sac gradually increases due to type II endoleaks, symptoms due to the mass effect are mainly present. Therefore, surgical removal of the aneurysm is helpful, rather than treatment of the endoleak through additional endovascular repair. However, in some cases, endovascular repair may be helpful in repairing the remaining fistula after surgery [8].

The goal of open surgical repair of subclavian artery aneurysm is to remove the aneurysm and maintain the continuity of blood flow. However, the subclavian artery is anatomically adjacent to the clavicle or sternum, making it difficult to fully expose the artery. Intrathoracic aneurysm is more easily accessible by sternotomy, and extrathoracic aneurysm requires access through the infra- or supra clavicular incision, and in some cases, clavicular resection. In our patient, the aneurysm originated in the proximal subclavian artery, but it was difficult to expose the entire aneurysmal sac. Therefore, to clamp the distal artery, it was necessary to extend the incision laterally or clavicular resection after sternotomy; however, by adding an additional small incision to the deltopectoral groove, the distal artery could be easily controlled.

Treatment of subclavian artery aneurysms has not been established; therefore, depending on the location and size of the aneurysm, it can be treated through endovascular repair or open surgery. Endovascular repair must be a method to reduce morbidity due to surgical incision, but since complications such as endoleak may occur in the long term, continuous follow-up is essential. If the aneurysm sac is large, surgical repair should be considered.

## Data Availability

Data sharing not applicable to this article as no datasets were generated or analysed during the current study.

## Abbreviations

CTA: Computed tomography angiography
VA: Vertebral artery
